# Supplementary material for: Eliminating chemo-mechanical degradation of lithium solid-state battery cathodes during >4.5 V cycling using amorphous Nb2O5 coatings
Source: Nat Commun. 2024 Nov 26;15:10233. doi: 10.1038/s41467-024-54331-w (PMC11599776; doi:10.1038/s41467-024-54331-w)
Supplement: Supplementary file 1 — Supplementary Information [file 41467_2024_54331_MOESM1_ESM.pdf]

Supporting information file

Eliminating chemo-mechanical degradation of lithium solid-state battery cathodes during >4.5V cycling using amorphous Nb_2O_5 coatings

Manoj K. Jangid [1,2#], Tae H. Cho [1,#], Tao Ma [3], Daniel W. Liao [1], Hwangsun Kim [2,4], Younggyu Kim [1,2], Miaofang Chi [2,4], Neil P. Dasgupta [1,2,5*]

[1] Department of Mechanical Engineering, University of Michigan, Ann Arbor, Michigan 48109, United States.

[2] MUSIC DOE Energy Frontier Research Center, University of Michigan, Ann Arbor, MI 48109, United States

[3] Michigan Center for Materials Characterization, University of Michigan, Ann Arbor, Michigan 48109, United States.

[4] Center for Nanophase Materials Sciences (CNMS), Oak Ridge National Laboratory, Oak Ridge, Tennessee 37831, United States.

[5] Department of Materials Science & Engineering, University of Michigan, Ann Arbor, Michigan 48109, United States.

[#] These authors contributed equally: Manoj K. Jangid, Tae H. Cho

[*] Corresponding author: ndasgupt@umich.edu

Table/ Figure	Description	Page No.
Table S1	XPS Elemental composition from uncoated and Nb_2O_5 coated powders	3
Figure S1	Rotary bed ALD process schematics	4
Figure S2	STEM-EDS near the (sub)surface of Nb_2O_5 coated NMC particle	5
Figure S3	XPS C *1s* core scan from Nb_2O_5-coated SC-NMC powder	6
Figure S4	Ni 2*p*, Mn 2*p* and Co 2*p* XPS scans from uncoated and Nb_2O_5-coated powders	7
Figure S5	Li 1*s*, Ni 3*p*, Mn 3*p*, Co 3*p* and Nb 4*s* XPS scans from uncoated and Nb_2O_5-coated NMC powders	8
Figure S6	XRD scans of uncoated and Nb_2O_5-coated NMC cathodes. XPS 3*d* and O 1*s* spectra of Nb_2O_5-coated NMC powder with and without sputtering	9
Figure S7	Cross-sectional view of a densified LTO|SE|NMC cell	10
Table S2	Theoretical capacities, actual capacities and corresponding weights of composite powders used for cell fabrication	11
Figure S8	Voltage profiles and corresponding dQ/dV plots at different cutoff voltages during the first formation cycle for uncoated and Nb_2O_5-coated SC-NMC cathodes	12
Table S3	Coulombic efficiency of uncoated and Nb_2O_5-coated SC-NMC cathode samples cycled to different cutoff voltage during the formation cycles	13
Figure S9	Voltage profiles at different C-rates for uncoated and Nb_2O_5 coated SC-NMC cathodes cycled at different cutoff voltages	14
Figure S10	Cyclic voltammogram of a Li|Li_6PS_5Cl|SS blocking electrode	15
Figure S11	Rate capability trends of uncoated, ALD Nb_2O_5-coated, and solution-processed $LiNbO_3$ coated SC-NMC composite cathodes at 4.3 V and 4.7 V voltage limits	16
Figure S12	Rate capability dQ/dV vs V plots of uncoated and Nb_2O_5-coated cathodes cycled at different voltage limits	17
Figure S13	Intermittent EIS measurement at different charging voltage points and total interfacial impedance for uncoated and Nb_2O_5-coated cathodes	18
Figure S14	Equivalent circuit used for fitting the Nyquist plots	19
Table S4	Fitted impedance components	19
Figure S15	GITT experiments and estimated internal resistance for uncoated and Nb_2O_5-coated cathodes	20
Figure S16	Coulombic efficiency of Nb_2O_5-coated SC-NMC cathodes at a 1C rate cycled with a 4.7 V limit using CC and CCCV protocols	21
Figure S17	*Ex-situ* XRD scans from uncoated and Nb_2O_5-coated SC-NMC cathodes before the cycling and after the rate capability tests	22
Figure S18	Intensity line profiles on TEM images of uncoated and Nb_2O_5-coated samples cycled for 500 cycles at a 1C rate with a 4.7 V limit	23
Figure S19	XPS core scans corresponding to Nb 3*d* peak and O 1*s* peak of Nb_2O_5-coated SC-NMC powders before and after cycling	24
Figure S20	FIB-SEM cross-section images of Nb_2O_5-coated SC-NMC electrodes before cycling and after 500 cycles tested at a 4.7 V limit	25
Table S5	Comparison of electrochemical performance of layered cathode materials having different coatings in solid electrolyte and liquid electrolyte systems	26
Table S6	List of abbreviations used in the study	28
	Supplementary References	29

Table S1: Elemental composition estimated by XPS on uncoated and Nb_2O_5 coated particle powders:

	Li	Ni	Mn	Co	Nb	O
Uncoat	17.64	2.99	2.14	1.14	-	36.79
Nb_2O_5	1.95	-	-	-	17.98	40.82

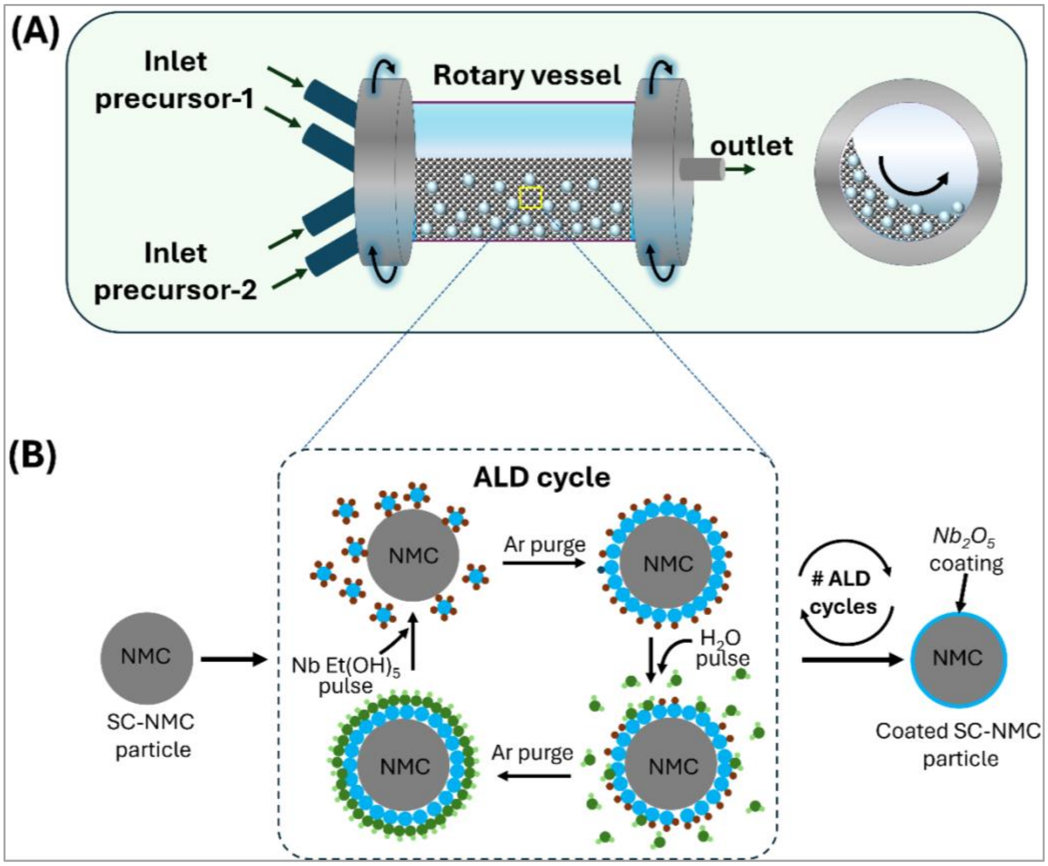

Figure S1: (A) Schematic of the ALD chamber with a rotary-bed attachment for conformal ALD coating at the particle scale on powders. (B) Schematic of the ALD process of Nb_2O_5 coating on an individual single-crystal NMC532 particle in the ALD chamber.

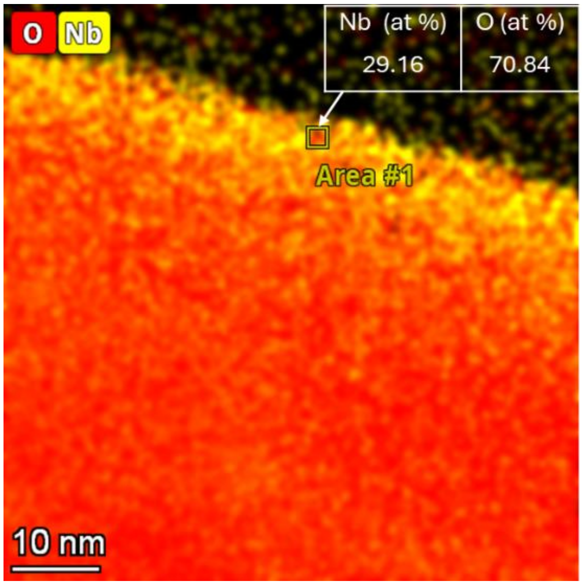

Figure S2: STEM-EDS elemental point scan showing Nb and O distributions near the (sub)surface of Nb_2O_5 coated NMC particle.

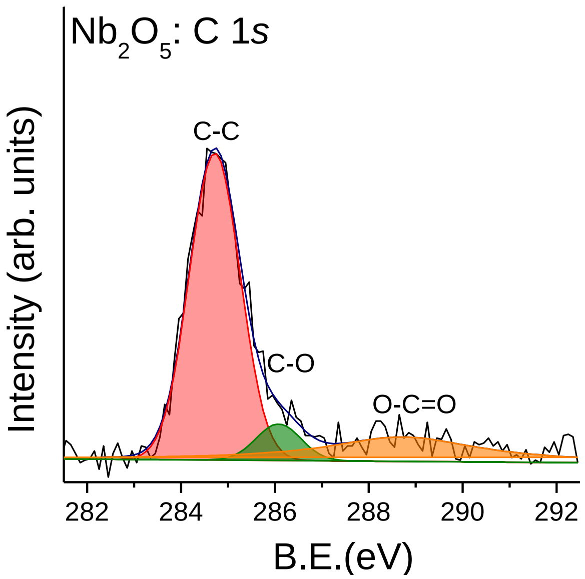

Figure S3: XPS C 1*s* core scan from Nb_2O_5-coated SC-NMC powder showing adventitious carbon.

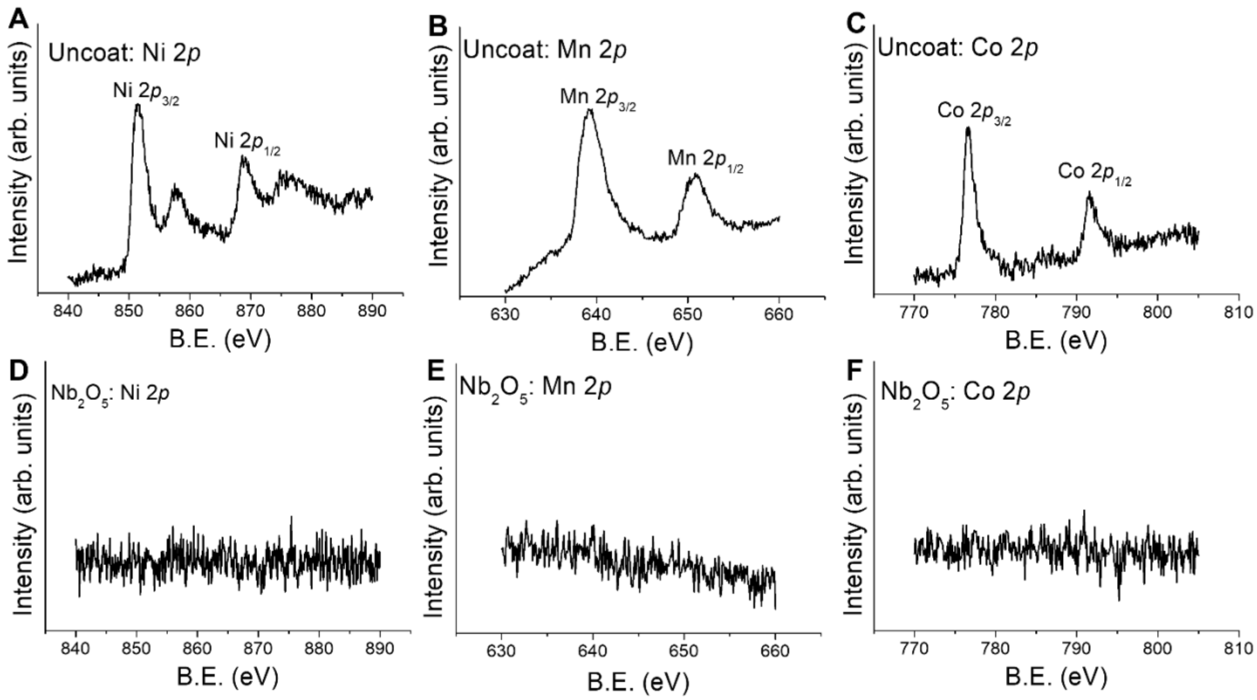

Figure S4: XPS core scans of Ni 2*p*, Mn 2*p* and Co 2*p* in (A-C) uncoated and (D-F) Nb_2O_5-coated SC-NMC powders.

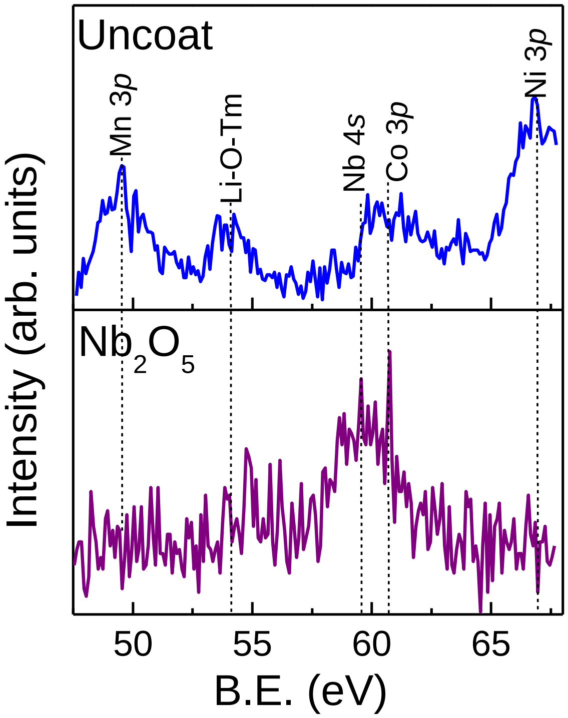

Figure S5: XPS Li 1*s*, Ni 3*p*, Mn 3*p*, Co 3*p* and Nb 4*s* core scans from uncoated and Nb_2O_5-coated NMC powders showing no evidence of $LiNbO_x$ formation in Nb_2O_5-coated NMC powder.

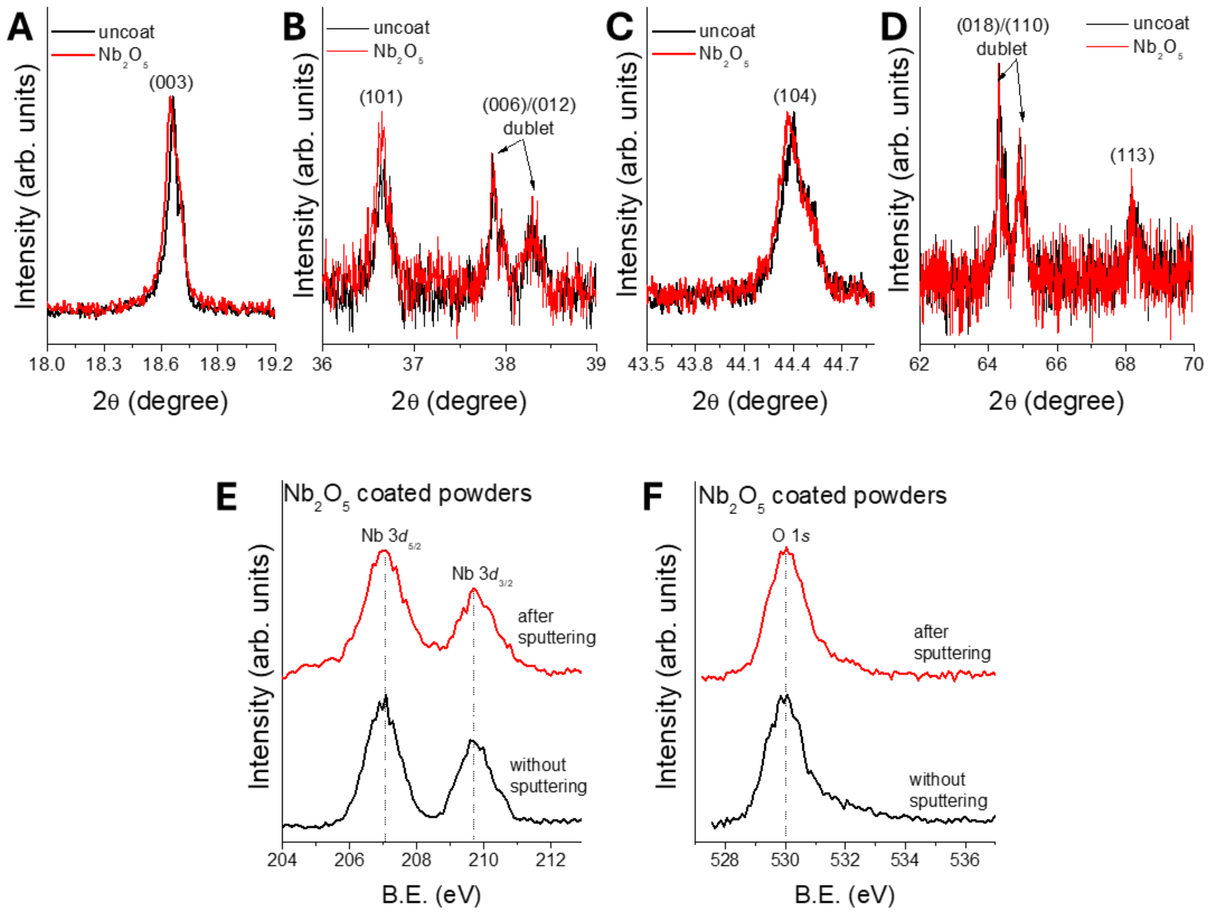

Figure S6: (A-D) Comparison of the positions of various peaks in XRD scans from uncoated and Nb_2O_5-coated NMC cathodes. (E-F) Comparison of the XPS 3*d* and O 1*s* spectra of Nb_2O_5-coated NMC powder with and without sputtering.

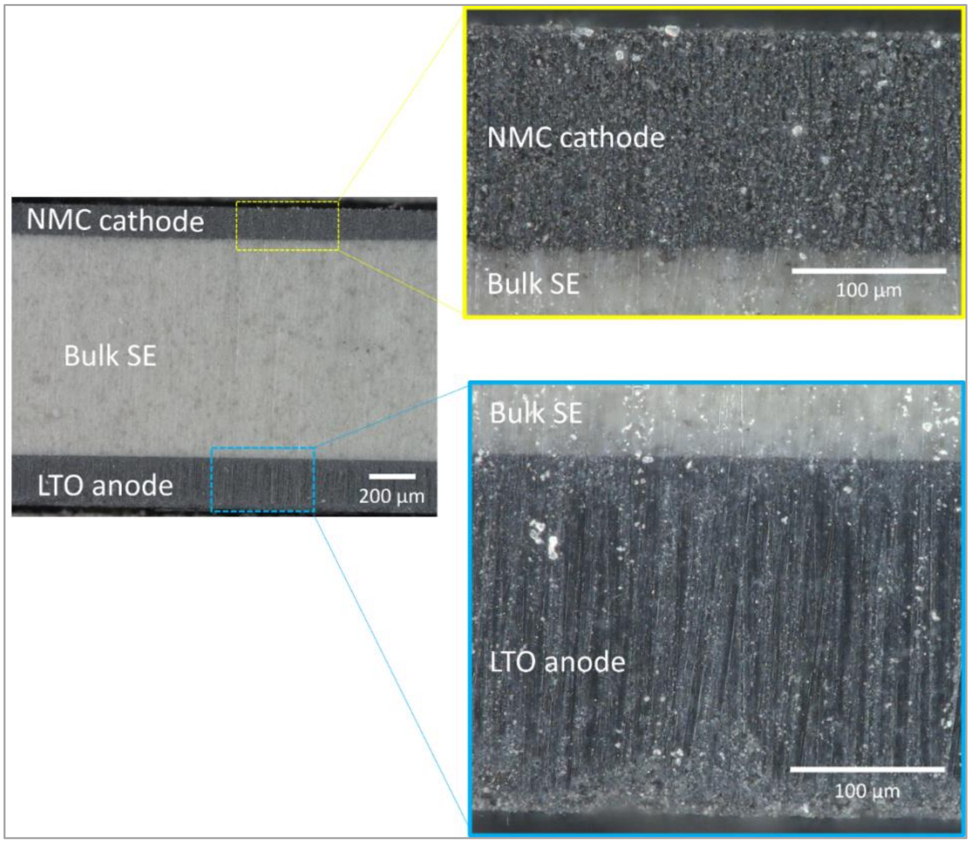

Figure S7: Cross-sectional view of a densified cell (LTO|SE|NMC pellet) with zoomed-in views of the anode (bottom) and cathode (top).

Table S2: Details of theoretical capacities considered in the study, actual capacities loaded in the electrodes and corresponding weights of composite powders used in composite electrodes:

Voltage range vs. LTO (vs. Li)	Theoretical accessible capacity ($mAh \cdot g^{-1}$)	Actual capacities ($mAh \cdot cm^{-2}$) of anode and cathode to maintain N/P= 1.1	Weight (g) of composite powders	
			anode	cathode
1.45-2.75 V (3.0-4.3 V)	SC-NMC =165<br>LTO = 155	Anode= 3.30<br>Cathode= 3.00	0.0137	0.0081
1.45-2.95 V (3.0-4.5 V)	SC-NMC =185<br>LTO = 155	Anode= 3.70<br>Cathode= 3.36	0.0149	0.0081
1.45-3.15 V (3.0-4.7 V)	SC-NMC =205<br>LTO = 155	Anode= 4.10<br>Cathode= 3.73	0.0163	0.0081

The theoretical capacity of NMC cathode considered in the present study were adopted from Zhang *et al.* (2020) [1] .

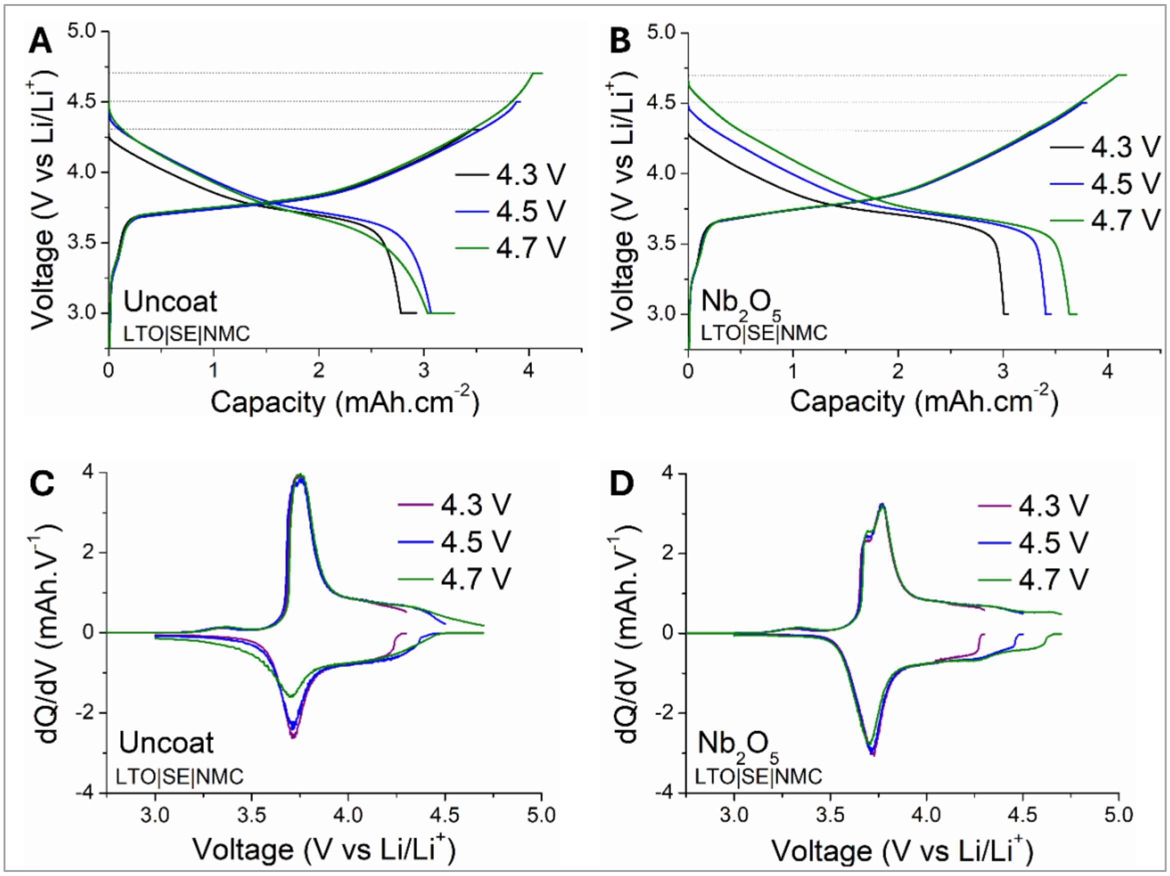

Figure S8: Voltage profiles and corresponding dQ/dV plots at different cutoff voltages during the first formation cycle for (A,C) uncoated and (B,D) Nb_2O_5-coated SC-NMC composite cathodes (LTO|SE|NMC), respectively.

Table S3: Coulombic efficiency of uncoated and Nb_2O_5-coated SC-NMC cathode samples (LTO|SE|NMC) cycled to different cutoff voltages during the formation cycles:

	**Formation cycle**	**Coulombic efficiency at different voltage limit (as %)**		
		**4.3 V**	**4.5 V**	**4.7 V**
Uncoat	1st	83.1	83.0	79.7
	2nd	98.2	97.6	97.1
	3rd	99.0	98.4	98.1
Nb_2O_5	1st	92.3	91.0	88.7
	2nd	98.9	99.5	97.9
	3rd	99.5	99.5	99.1

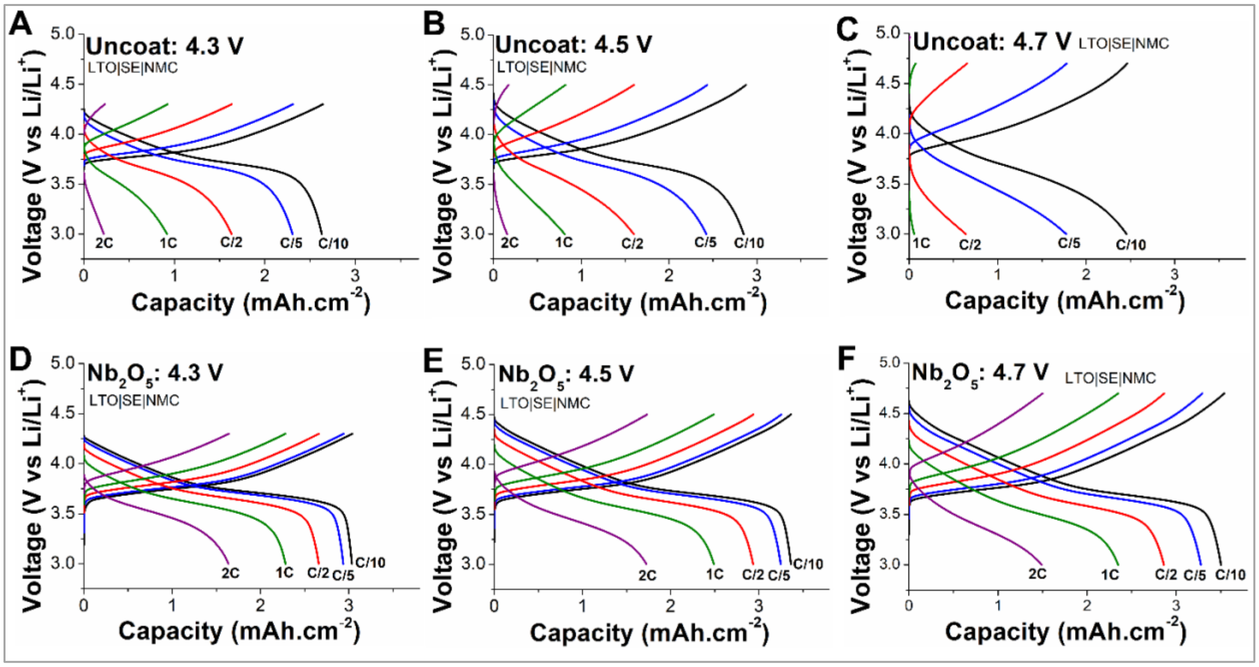

Figure S9: Voltage profiles at different C-rates (C/10, C/5, C/2, 1C, 2C) for (A-C) uncoated and (D-F) Nb_2O_5 coated SC-NMC composite cathodes (LTO|SE|NMC) cycled at different cutoff voltages.

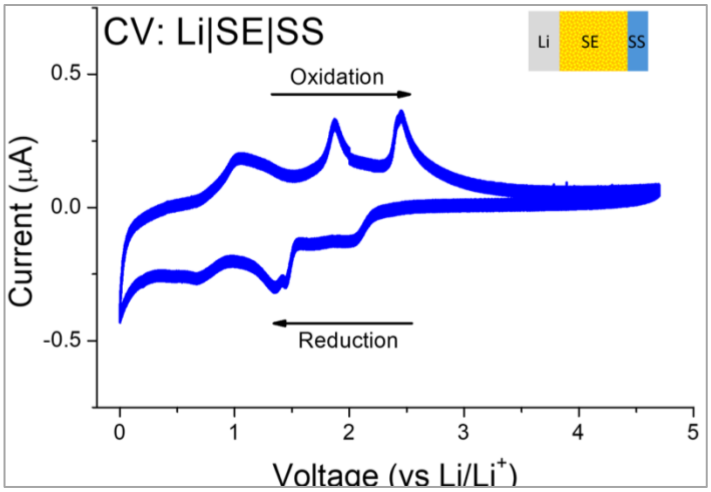

Figure S10: Cyclic voltammogram of a Li|Li_6PS_5Cl|SS blocking electrode at a 0.1 mV·s^{-1} scan rate between 0 and 4.7 V (vs Li/Li^+) showing instability and degradation of Li_6PS_5Cl solid electrolyte.

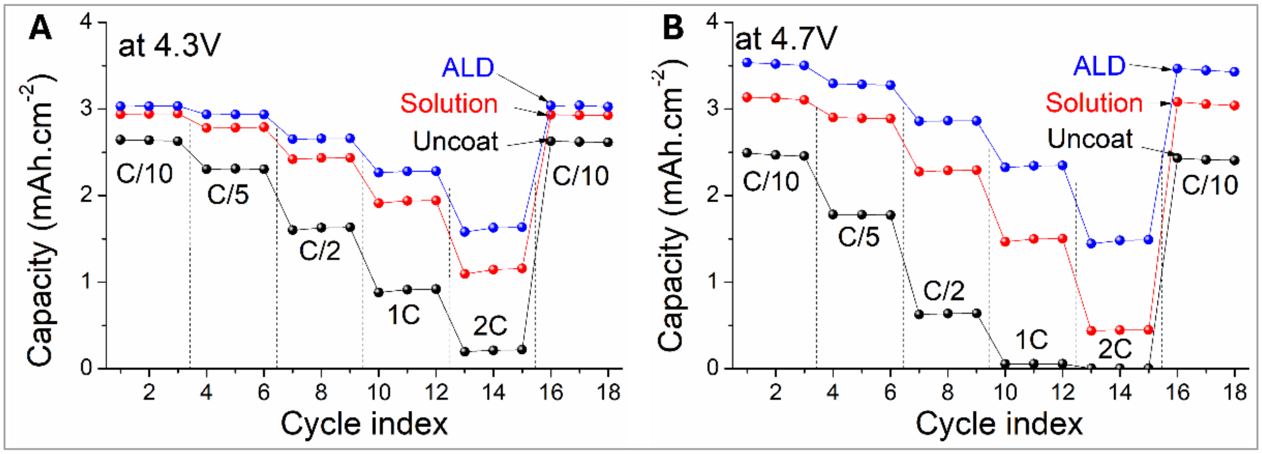

Figure S11: Rate capability trends of uncoated, ALD Nb_2O_5-coated, and solution-processed $LiNbO_3$ coated SC-NMC composite cathodes (LTO|SE|NMC) at (A) 4.3 V and (B) 4.7 V cutoff voltages.

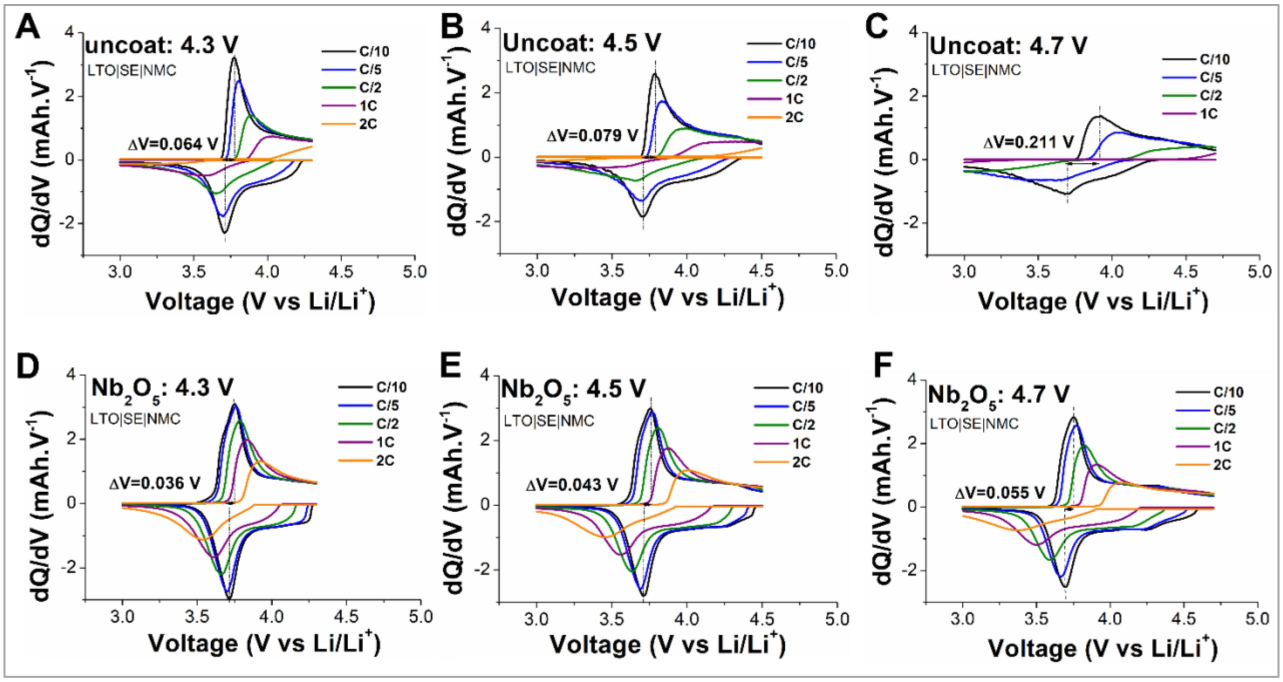

Figure S12: dQ/dV vs V plots obtained from the voltage profiles during rate capability tests of (A-C) uncoated and (D-F*)* Nb_2O_5-coated SC-NMC composite cathodes (LTO|SE|NMC) cycled at different cutoff voltages.

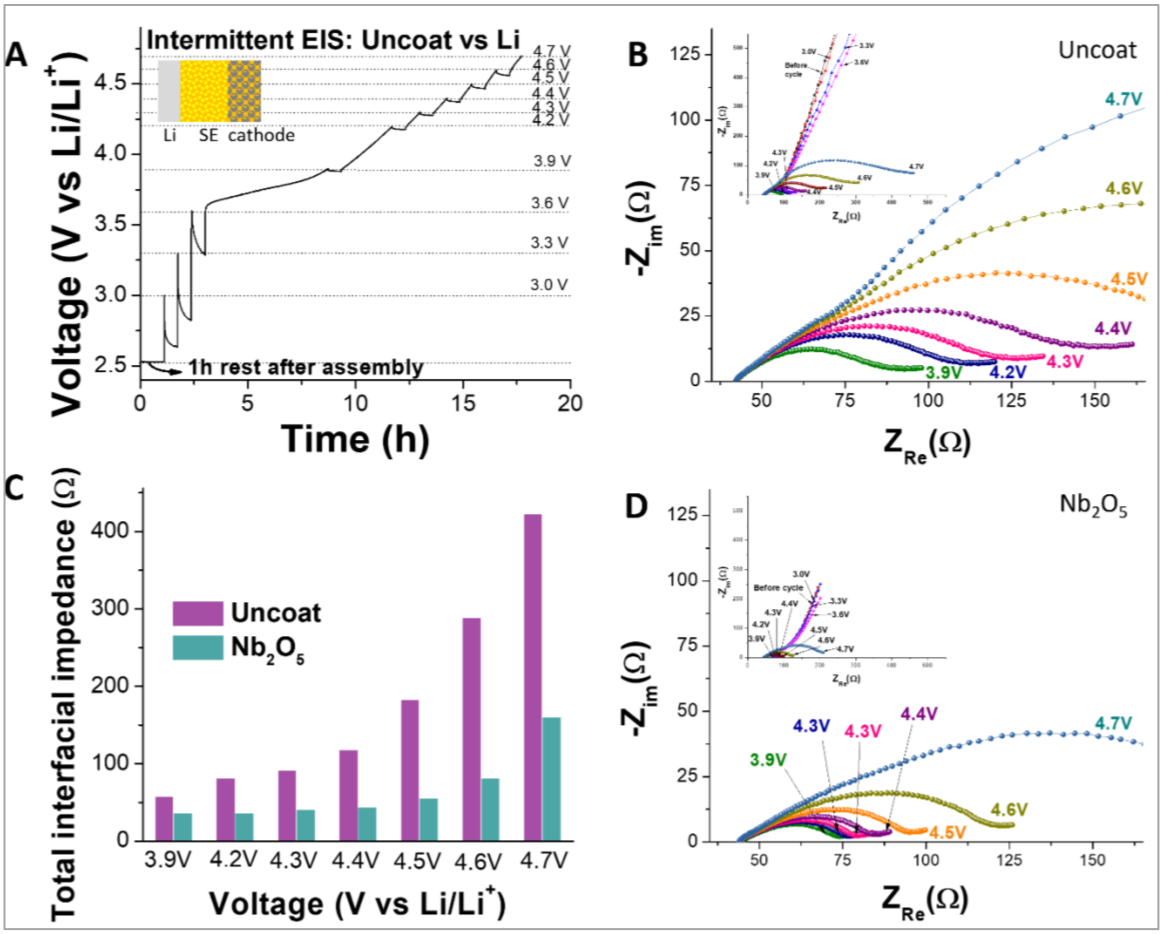

Figure S13: (A) Intermittent EIS measurement at specific voltage points during the charging cycle (at C/10 rate) of uncoated and Nb_2O_5-coated cathodes (Li|SE|NMC). Zoomed-in view of Nyquist plots obtained at different voltage points during the charging cycle are presented for (B) uncoated and (D) Nb_2O_5-coated cathodes, with insets providing a zoomed-out view of the Nyquist plots. (C) Comparison of total interfacial impedance at different voltage points during charging.

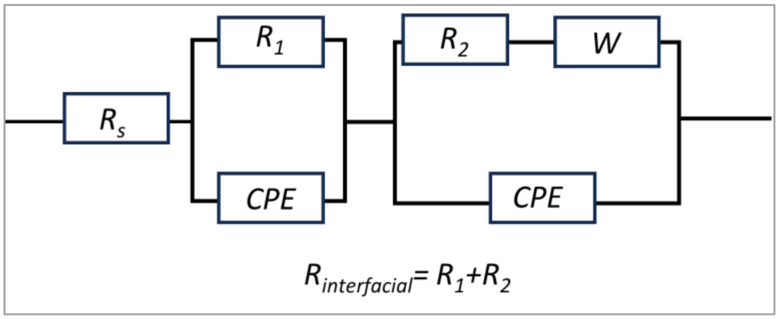

Figure S14: Equivalent circuit used for fitting the Nyquist impedance plots.

Table S4: Fitted impedance components:

	Uncoat				Nb_2O_5			
Voltage limit	Rs (Ω)	R_1 (Ω)	R_2 (Ω)	$R_{Interfacial}$ (Ω) (R_1+R_2)	Rs (Ω)	R_1 (Ω)	R_2 (Ω)	$R_{Interfacial}$ (Ω) (R_1+R_2)
4.3 V	49.2	133.4	366.2	499.6	46.9	32.9	54.7	87.5
4.5 V	47.1	289.1	356.5	654.6	60.0	28.6	56.9	85.3
4.7 V	51.3	547.3	805.0	1352.4	43.3	84.6	74.5	159.1

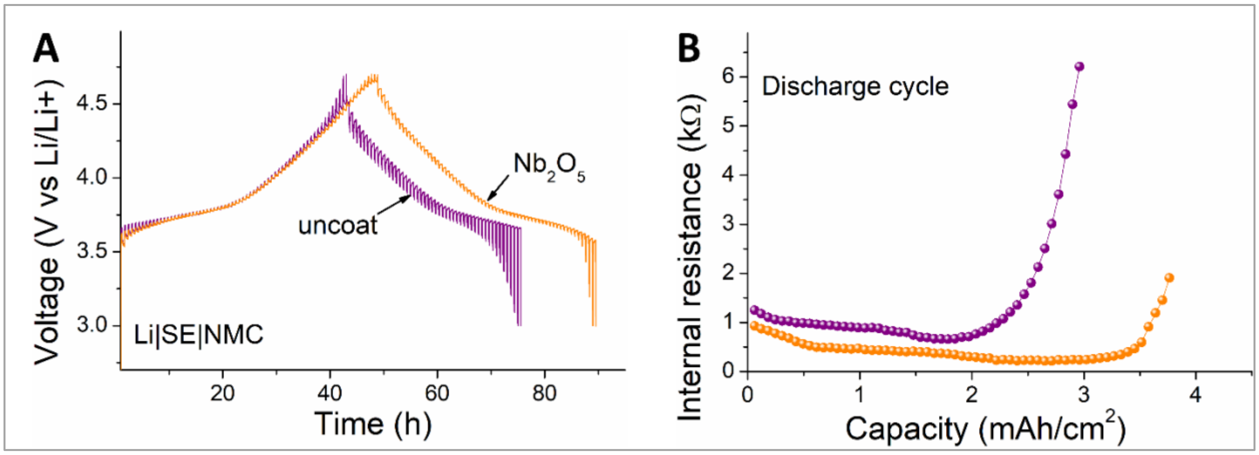

Figure S15: (A) Voltage trends during GITT experiments of uncoated and Nb_2O_5-coated cathodes (Li|SE|NMC). (B) Internal resistance during discharge half cycles estimated from the voltage profiles obtained during the GITT experiments. The GITT experiments were performed with Li|SE|NMC cells by applying a current pulse equivalent to C/10 rate for 10 min followed by a 30 min open circuit rest.

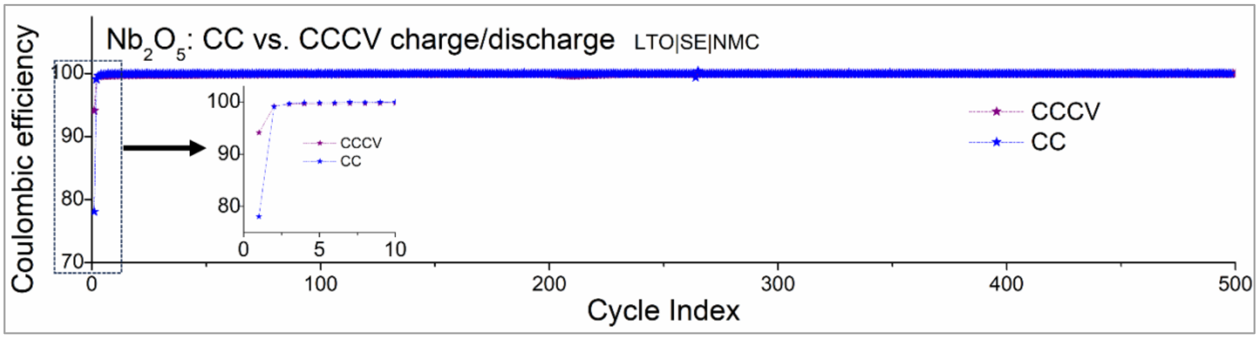

Figure S16: (A) Coulombic efficiency of Nb_2O_5-coated SC-NMC cathodes (LTO|SE|NMC) at 1C rate cycled with a 4.7 V limit using CC and CCCV charge/discharge protocols. (Inset) Coulombic efficiency during the first 10 cycles.

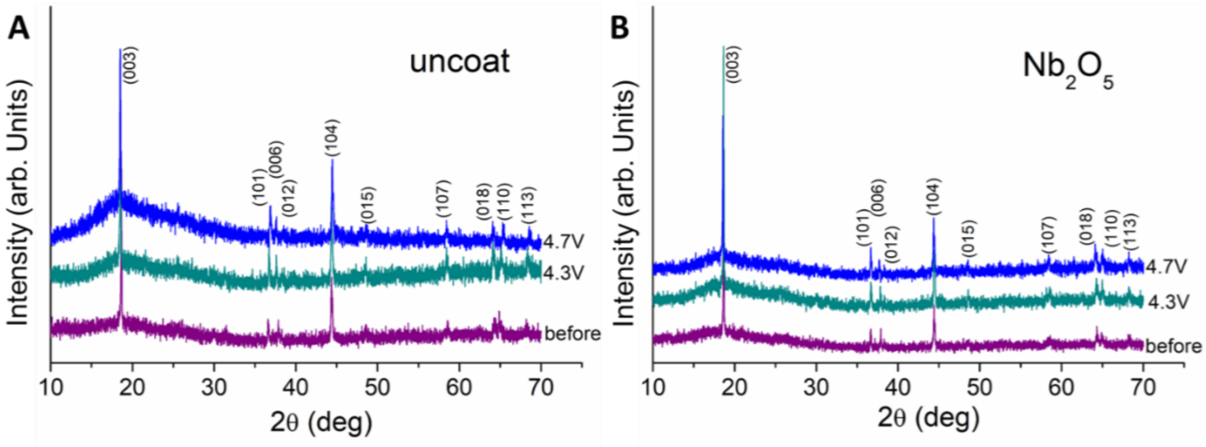

Figure S17: *Ex-situ* XRD scans of recorded at the cathode sides of the pressed LTO|SE|NMC cells before cycling and after the rate capability tests performed with 4.3 V and 4.7 V limits for (A) uncoated and (B) Nb_2O_5-coated SC-NMC samples.

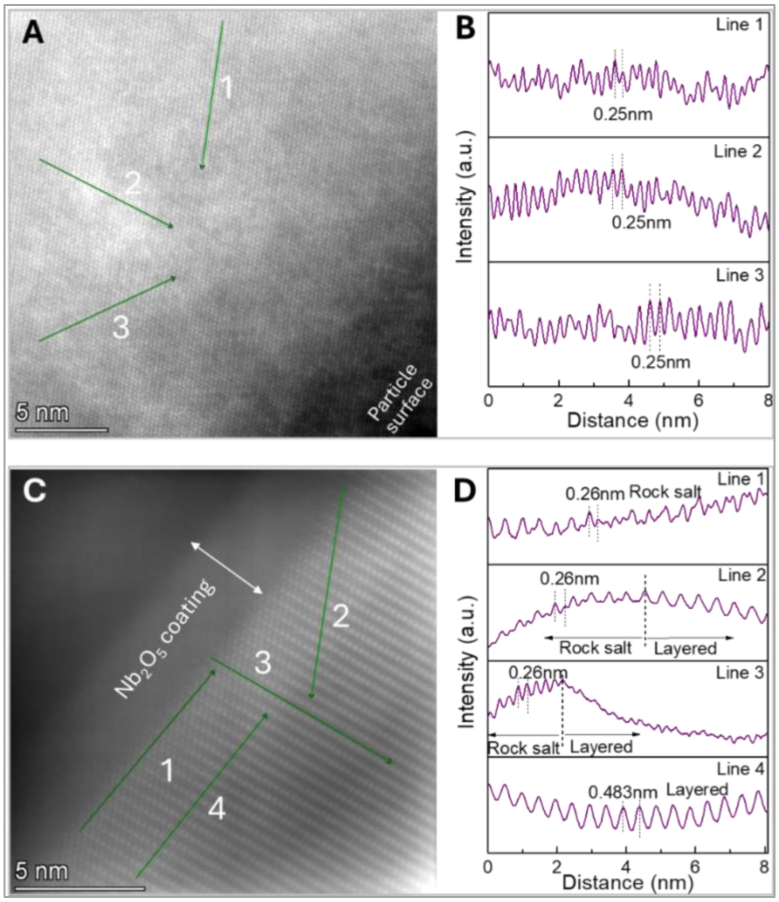

Figure S18: Intensity line profiles on TEM images obtained from (A,B) uncoated and (C,D) Nb_2O_5-coated samples (LTO|SE|NMC) cycled for 500 cycles at 1C and 4.7 V showing interplanar spacing along different crystallographic directions.

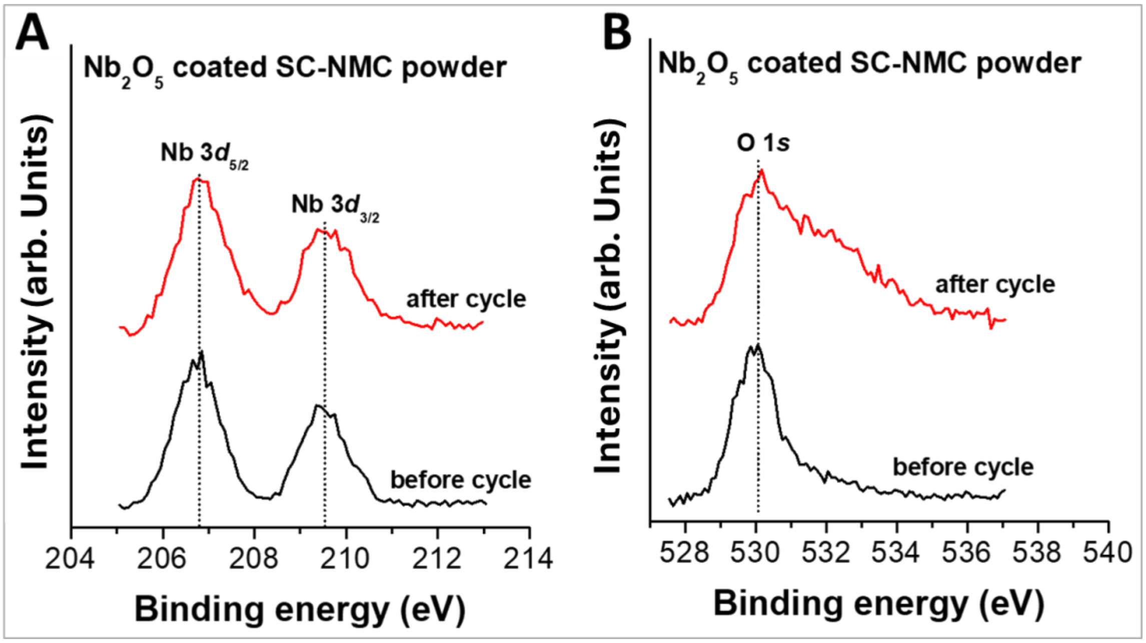

Figure S19: XPS core scans corresponding to (A) Nb *3d* peak (B) O *1s* peak from Nb_2O_5-coated SC-NMC powders before cycling and after cycling.

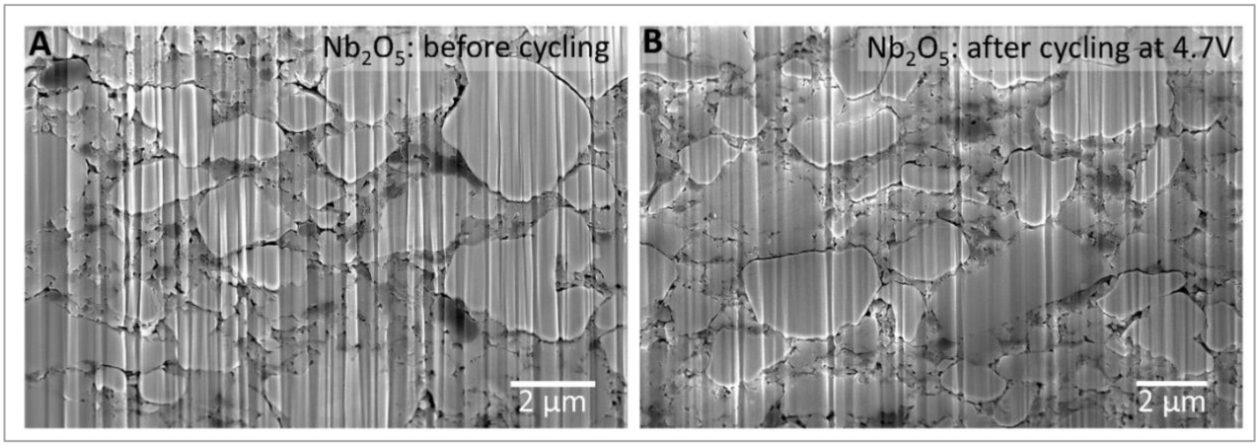

Figure S20: FIB-SEM cross-section images of Nb_2O_5-coated SC-NMC electrodes (A) before cycling and (B) after 500 cycles (LTO|SE|NMC) tested at 1C rate with a 4.7 V limit.

Table S5: Comparison of electrochemical performance of layered cathode materials having different coatings in solid electrolyte and liquid electrolyte systems.

Coating material	Coating Thick-ness	Coating method	Cathode material	Electro-lyte	Anode	Upper Volt-age limit (V vs Li/Li^+)	1st cycle CE (Voltage limit, current density)	Capacity retention as % (voltage limit, C-rate, after cycles)	Ref
Al_2O_3	0.4-1.4 nm	ALD	$LiNi_{0.5}Mn_{1.5}O_4$	Li_6PS_5Cl	Li-In	4.4V (5.0V)	86.5% (5.0V,	70.1% (5.0V, 0.2C, 100 cycles)	2
ZrO_2	4-5 nm	ALD	$LiNi_{0.85}Co_{0.1}Mn_{0.5}O_2$	Li_6PS_5Cl	LTO	2.75V (4.3V)	~91% (4.3V, 0.1C)	78% (4.3V, 0.5C, 200 cycles)	3
HfO_2	2-3nm	ALD	$LiNi_{0.85}Co_{0.1}Mn_{0.5}O_2$	Li_6PS_5Cl	Li	4.3V	~88% (4.3V, 0.1C)	82% (4.3V, 0.5C, 60 cycles)	4
$LiNbO_3$	2-5 nm	Solution	$LiNi_{0.82}Co_{0.12}Mn_{0.6}O_2$	Li_6PS_5Cl	Li-In	3.7V (4.32) 3.9V (4.52 V)	71.8% (4.32V, 8.5 mA/g)	82.1% (4.52V, 34 mA/g, 30 cycles)	5
$LiNbO_3$	10-20nm	Solution	$LiNi_{0.5}Mn_{1.5}O_4$	Li_6PS_5Cl / Li_3YCl_6	Li-In	4.25V (4.85 V)	91.2% (4.85V, 7.5 mA/g)	~50% (4.85V, 20mA/g, 50 cycles)	6
$LiNbO_x$	4 nm	ALD	$LiNi_{0.8}Co_{0.1}Mn_{0.1}O_2$	$Li_{10}GeP_2S_{12}$	LTO	2.8V (4.35 V)	80.6% (4.35V, 0.1C)	76.3% (4.35V, 0.3C, 400 cycles)	7
Li_3BO_3	1-11 nm	Solution	$LiCoO_2$	Li_6PS_5Cl	Li-In	3.68V (4.3V) 3.88V (4.5V)	91% (4.3V)	88.7% (4.5V, 0.2C, 25cycles)	8
Li_3BO_3–Li_2CO_3	21-30 nm	Solution	$LiCoO_2$	Li_6PS_5Cl	Li-In	3.68V (4.3V) 3.88V (4.5V)	93% (4.3V)	93.8% (4.5V, 0.2C, 25cycles)	8
$LiTaO_x$	2-6 nm	Solution	$LiNi_{0.82}Co_{0.12}Mn_{0.6}O_2$	Li_6PS_5Cl	Li-In	3.7V (4.32) 3.9V (4.52 V)	76.1% (4.32V, 8.5 mA/g)	83% (4.52V, 30 cycles)	5
Li_3PO_4	1-10 nm	ALD	$LiNi_{0.8}Co_{0.1}Mn_{0.1}O_2$	$Li_{10}GeP_2S_{12}$	In	3.88V (4.5V)	75.1% (4.5V, 0.1C)	78% (4.4V, 0.2C, 100 cycles)	9
Li_2ZrO_3	<10 nm	Solution	$LiNi_{0.82}Co_{0.12}Mn_{0.6}O_2$	Li_6PS_5Cl	LTO	2.85V (4.4V)	86% (4.4V, 0.2C)	~70% (4.4V, 0.1C, 60 cycles)	10
$LiWO_3$	2-4 nm	Solution	$LiNi_{0.6}Co_{0.2}Mn_{0.2}O_2$	$75Li_2S$–$22P_2S_5$–$3Li_2SO_4$	Li-In	3.88V (4.5V)	64.4% (4.5V, 0.05C)	83% (4.5V, 0.1C, 30 cycles)	11
$Li_xAl_yZn_zO_\delta$	~4 nm	ALD	$LiNiO_2$	Li_6PS_5Cl	Li-In	4.3V	85.4% (4.3V, 0.2C)	83.1% (4.3V,	12

								0.2C, 200 cycles)	
$LiAl(PO_3)_4$	4nm	ALD	$LiNi_{0.88}Co_{0.09}Mn_{0.03}O_2$	Li_6PS_5Cl	Li-In	4.3V	84.1%, 4.3V, C/5	98.3%, 440 cycles 20.1 mg/cm2	[13]
CoO/Li_2CO_3	4nm	Heat treatment	$LiCoO_2$	Li_6PS_5Cl	Li-In	4.6V	83%, 4.3V, C/2	83%, 150 cycles, C/2	[14]
Gd_2O_3	7 nm	Solution	$LiNi_{0.6}Co_{0.05}Mn_{0.35}O_2$	Liquid	Li	4.5V	~83% (4.5V, 0.1C)	88.1% (4.5V, 1C, 400 cycles)	[15]
Sm_2O_3	13 nm	Solution	$LiNi_{0.6}Co_{0.05}Mn_{0.35}O_2$	Liquid	Li	4.5	~82% (4.5V, 0.1C)	97.0 % (4.5V, 1C, 300 cycles)	[16]
Al_2O_3	1-4 nm	ALD	$LiNi_{0.6}Co_{0.2}Mn_{0.2}O_2$	Liquid	Li	4.7V	~85%(4.7V, 0.5C)	89.5% (4.7V, 0.5C, 45 cycles)	[17]
ZrO_2	Not available	Ball mill	$Li_{1.2}Ni_{0.13}Co_{0.13}Mn_{0.54}O_2$	Liquid	Li	4.8V	82.5 (4.8V, 0.1C)	89.0% (4.8V, 1C, 100 cycles)	[18]
Li_3PO_4	20 nm	Solution	$LiCoO_2$	Liquid	Li	4.5V	87.3% (4.5V, 0.1C)	90% (4.5V, 0.5C, 100 cycles)	[19]
AlZnO	3nm	solution	$LiCoO_2$	Liquid	Li	4.6V	~82% (4.6V, 37 mA/g	65.7% (4.6V, 185 mA/g, 500 cycles	[20]
$Li_{0.5}Mn_{0.5}O$		solution	$Li_{1.2}Mn_{0.6}Ni_{0.2}O_2$	Liquid	Li	4.8V	80.3% (4.8V, C/10)	80.7% after 200 cycles at 1 C	[21]

Table S6: List of abbreviations used in the study

SSB: Solid-state battery	SEI: Solid electrolyte interface
LIB: Li-ion battery	ASR: Area-specific resistance
EV: Electric vehicle	XPS: X-ray photoelectron spectroscopy
SE: Solid electrolyte	FIB: Focused ion beam
LE: Liquid electrolyte	TEM: Transmission electron microscopy
CAM: Cathode active material	STEM: Scanning transmission electron microscopy
NMC: Nickel manganese cobalt oxide	EDS: Electron dispersive X-ray spectroscopy
SC: Single crystal	HAADF: High-angle annular dark-field
LPSC: Li_6PS_5Cl	XRD: X-ray diffraction
ALD: Atomic layer deposition	EIS: Electrochemical impedance spectroscopy
TM: Transition metal	GITT: Galvanostatic intermittent titration technique
PTFE: Polytetrafluoroethylene	FFT: Fast Fourier transform
LTO: Lithium titanate	CC: Constant current
CEI: Cathode electrolyte interface	CV: Constant voltage
ICE: Initial Coulombic efficiency	CCCV: Constant current constant voltage

Supplementary References:

1. Zhang, L., Zhang, C., Li, N. & Tong, W. Influence of Charge Cutoff Voltage on the Cycling Behavior of LiNi 0.5 Mn 0.3 Co 0.2 O 2 Cathode. *J Electrochem Soc* **167**, 120509 (2020).

2. Lee, H. J. *et al.* LiNi0.5Mn1.5O4Cathode Microstructure for All-Solid-State Batteries. *Nano Lett* **22**, 7477–7483 (2022).

3. Kitsche, D. *et al.* Atomic Layer Deposition Derived Zirconia Coatings on Ni-Rich Cathodes in Solid-State Batteries: Correlation Between Surface Constitution and Cycling Performance. *Small Science* **3**, 1–12 (2023).

4. Kitsche, D. *et al.* High Performance All-Solid-State Batteries with a Ni-Rich NCM Cathode Coated by Atomic Layer Deposition and Lithium Thiophosphate Solid Electrolyte. *ACS Appl Energy Mater* **4**, 7338–7345 (2021).

5. Lee, J. S. & Park, Y. J. Comparison of $LiTaO_3$and $LiNbO_3$ Surface Layers Prepared by Post- and Precursor-Based Coating Methods for Ni-Rich Cathodes of All-Solid-State Batteries. *ACS Appl Mater Interfaces* **13**, 38333-38345 (2021).

6. Jang, J. *et al.* Enabling a Co-Free, High-Voltage $LiNi_{0.5}Mn_{1.5}O_4$ Cathode in All-Solid-State Batteries with a Halide Electrolyte. *ACS Energy Lett* **7**, 2531–2539 (2022).

7. Liu, X. *et al.* Constructing a High-Energy and Durable Single-Crystal NCM811 Cathode for All-Solid-State Batteries by a Surface Engineering Strategy. *ACS Appl Mater Interfaces* **13**, 41669–41679 (2021).

8. Jung, S. H. *et al.* Li_3BO_3-Li_2CO_3: Rationally Designed Buffering Phase for Sulfide All-Solid-State Li-Ion Batteries. *Chem Mater* **30**, 8190–8200 (2018).

9. Deng, S. *et al.* Dual-functional Interfaces for Highly Stable Ni-Rich Layered Cathodes in Sulfide All-Solid-State Batteries. *Energy Storage Mater* **27**, 117–123 (2020).

10. Strauss, F. *et al.* $LiZrO_3$-coated NCM622 for Application in Inorganic Solid-State Batteries: Role of Surface Carbonates in the Cycling Performance. *ACS Appl Mater Interfaces* **12**, 57146–57154 (2020).

11. Lim, C. B. & Park, Y. J. Precursor-based Surface Modification of Cathodes using Ta and W for Sulfide-Based All-Solid-State Batteries. *Sci Rep* **10**, 1–12 (2020).

12. Wang, L. *et al.* High-energy All-Solid-State Lithium Batteries Enabled by Co-Free $LiNiO_2$ Cathodes with Robust Outside-in Structures. *Nat Nanotechnol* **19**, 208–218 (2024).

13. Zhou, X. *et al.* High-Performance Sulfide All-Solid-State Batteries Enabled by High-Voltage Ni-Rich Cathode with a Conformal and Conductive Protective Layer. *ACS Appl Energy Mater* **7**, 2524–2532 (2024).

14. Zheng, S. *et al.* Surface Reconstruction Layer Boosting Interfacial Stability of $LiCoO_2$/Li_6PS_5Cl in Bulk All-Solid-State Li Batteries. *J Mater Chem A Mater* **12**, 7916–7922 (2024).

15. Shen, Y. *et al.* A Universal Multifunctional Rare Earth Oxide Coating to Stabilize High-Voltage Lithium Layered Oxide Cathodes. *Energy Storage Mater* **56**, 155–164 (2023).

16. Shen, Y. *et al.* Stabilization of High-Voltage Layered Oxide Cathode by Multi-Electron Rare Earth Oxide. *Chem Eng J* **454**, 140249 (2023).

17. Wang, X., Cai, J., Liu, Y., Han, X. & Ren, Y. Atomic-scale Constituting Stable Interface. *Nanotechnology* **32**, 115401 (2021).

18. Cao, J. *et al.* Enhanced Electrochemical Performances of $Li_{1.2}Ni_{0.13}Co_{0.13}Mn_{0.54}O_2$ Cathode Material Coated with ZrO_2. *Ionics* **29**, 51–60 (2023).

19. Ji, Y. *et al.* $LiCoO_2$ Cathode Surface Modification with Optimally Structured Li_3PO_4 for Outstanding High-Voltage Cycling Performance. *Nanoscale* **15**, 11898–11908 (2023).

20. Cheng, T. *et al.* Achieving Stable Cycling of $LiCoO_2$ at 4.6 V by Multilayer Surface Modification. *Adv Funct Mater* **31**, 1–8 (2021).

21. Zhu, A. *et al.* In-situ formation of $Li_{0.5}Mn_{0.5}O$ Coating Layer Through Defect Controlling for High Performance Li-Rich Manganese-Based Cathode Material. *J Energy Chem* **71**, 384–391 (2022).
